# The curious case of the crystalline tri-thorium cluster: cyclic delocalization without aromatic stabilization?

**DOI:** 10.1039/d3ra06603g

**Published:** 2023-11-22

**Authors:** Dariusz W. Szczepanik

**Affiliations:** a Department of Theoretical Chemistry, Faculty of Chemistry, Jagiellonian University Gronostajowa, 2 30-387 Kraków Poland dariusz.szczepanik@uj.edu.pl

## Abstract

Actinides have been known to form extremely weak homonuclear bonds with their d-type orbitals, and one should therefore expect the superposition of cyclic resonance forms containing such bonds to bring rather marginal aromatic stabilization to the system, if any. It is for this very reason that the discovery of the cyclically delocalized Th_3_ σ-bonding in the crystalline cluster isolated by Liddle and co-workers has sparked such vigorous discussion on the actual role of molecular aromaticity on the periphery of the periodic table. It has recently been argued that the tri-thorium ring at the heart of the cluster features considerable aromatic stabilization energy comparable to the heterocyclic π-aromatic rings such as thiophene. However, previous investigations involved highly ionized model clusters like Th_3_Cl_6_^4+^ or Th_3_^10+^ in which aromatic stabilization associated with the cyclic delocalization of charge is dramatically exaggerated. In this work we investigate the model tri-thorium clusters at different geometries and ionization states to show that cyclic delocalization of electrons in the isolated crystalline cluster may be associated with rather marginal σ-aromatic stabilization energy thus strongly suggesting its non-aromatic character.

## Abstract 2

Actinides have been known to form weak homonuclear bonds with their d-type orbitals, and thus one should expect the superposition of cyclic resonance forms containing such bonds to bring rather marginal aromatic stabilization to the system, if any.

## Graphical abstract

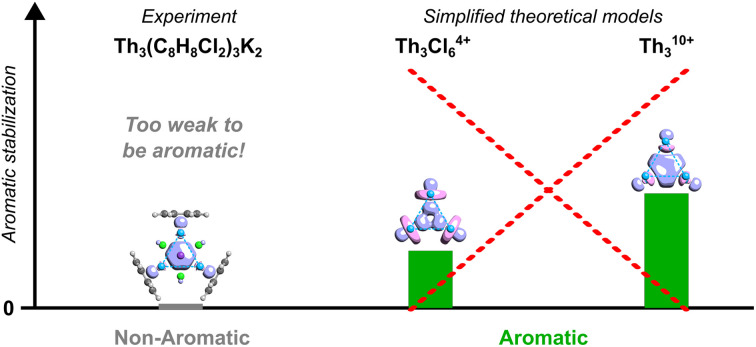

## Introduction

When Kekulé first pictured the cyclic structure of benzene,^1^ hardly anyone could have imagined that electron delocalization and aromaticity may play a central role in an incredible variety of inorganic rings and clusters containing s-, d- and even f-block metal atoms.^2^ Very recently, Liddle and co-workers isolated under normal experimental conditions the crystalline actinide cluster [{Th(η^8^-C_8_H_8_)(μ^3^-Cl)_2_}_3_{K(THF)_2_}_2_]_∞_ containing at its heart the Th_3_ ring with a pair of uniformly distributed electrons.^3^ This discovery has been hailed for extending the range of aromaticity to the record seventh row of the periodic table. Also, it has been suggested that aromaticity can be an important factor in the design of stable complexes with actinide–actinide bonds, which have previously been reported in the literature to be weak and localized.^4^ However, the follow-up investigations revealed that both experimental and computational data (especially results of aromaticity assessment based on the magnetic criteria)^5^ may not provide solid proof for the existence of the aromatic Th_3_ bonding^6,7^ and the unique multicenter (delocalized) charge-shift bonding (ThCl_2_)_3_ was shown to be the actual factor determining high symmetry (*D*_3h_) and exceptional thermodynamical stability of the crystalline actinide cluster.^7^ Lin and Mo provided further computational data supporting the charge-shift bonding in the actinide cluster.^8^ Moreover, the authors found that the overall charge transfer between Th and Cl atoms is effectively ten times more stabilizing than the electron delocalization in the Th_3_ ring.^8^ Lin and Mo linked the latter with the stabilizing effect resulting from the periodic boundary conditions (PBC) for the electron distribution,^9^ and made the conclusion that the Th_3_ bonding is “truly delocalized and σ-aromatic”.^8^ Similar conclusion was recently drawn by Tomeček *et al.*,^10^ who utilized the entire arsenal of different sophisticated computational methods to demonstrate that Th_3_ contains cyclically delocalized electrons and should be regarded σ-aromatic. In both works,^8,10^ however, the simplified ionized model cluster Th_3_Cl_6_^4+^ was investigated, in which electron binding energy associated with the highest occupied molecular orbital (HOMO) is incomparably higher in magnitude than in the neutral crystalline cluster. This leads to dramatic exaggeration of σ-bonding between thorium atoms (a direct manifestation of which is contraction of the Th–Th bond length by about 0.3 Å) and makes the conclusions by Lin and Mo,^8^ and Tomeček *et al.*,^10^ not directly transferable to the Th_3_ core found in the experimentally isolated actinide cluster.^3^

In this work we show how separation of charge in the ionized model tri-thorium clusters investigated previously by different authors affects aromaticity predictions. Furthermore, we demonstrate that the tri-thorium σ-bonding in the cyclic isomer brings rather marginal extra stabilization compared to the linear one, which suggests non-aromatic character in accordance with the IUPAC recommendation.^11^

## The covalent resonance energy issue in Th_3_

**Fig. 1:**
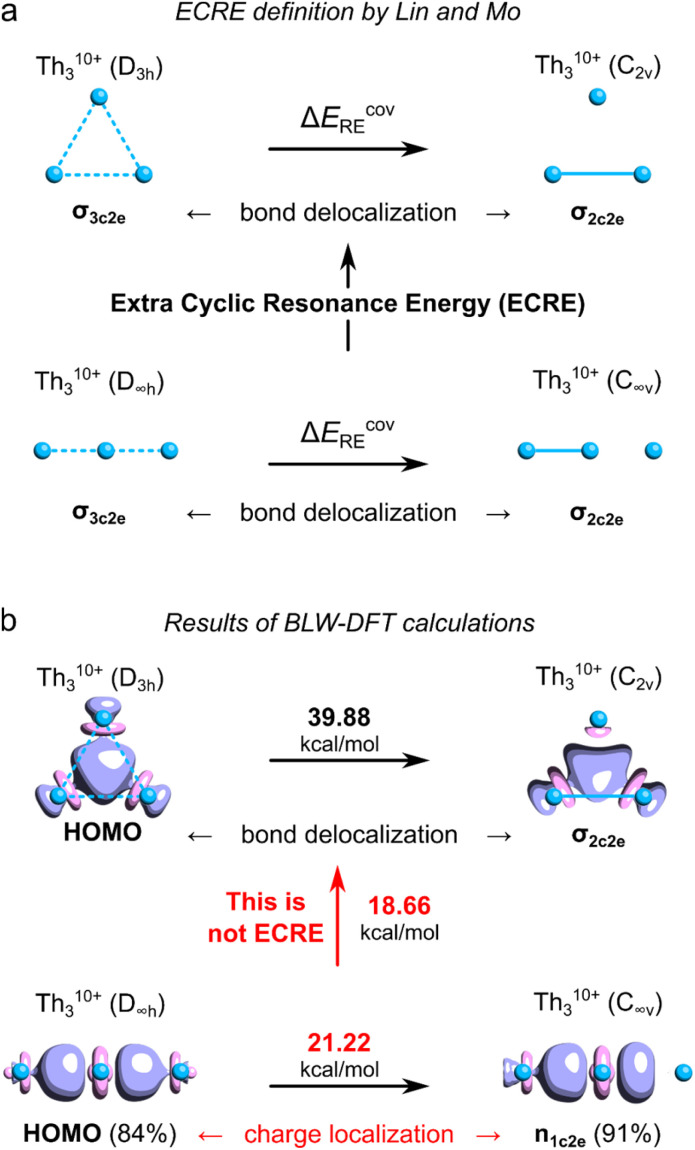
(a) Definition of the ECRE for Th_3_^10+^ proposed by Lin and Mo. (b) The results of the BLW-DFT based calculations of ECRE with the corresponding HOMO isosurfaces (visualized at *τ* = 0.07*e*). Numbers in brackets represent degree of charge localization based on the molecular orbital coefficients. The ECRE calculations were performed using the same methods and software as used in the original work by Lin and Mo.^8^

To quantitatively assess aromatic stabilization in the Th_3_ core of the isolated crystalline cluster, Lin and Mo utilized the highly ionized model Th_3_^10+^ derived from the Th_3_Cl_6_^4+^ cage at its equilibrium geometry (with the Th–Th bond length of 3.684 Å, thus being much shorter from the experimentally measured 3.991 Å).^8^ In both highly charged models, the highest-occupied molecular orbitals (HOMO) visually resemble the corresponding HOMO in the original model cluster investigated by Liddle and co-workers.^3^ The authors used a sophisticated computational method called the block-localized wavefunction (BLW) within the framework of the density functional theory (DFT)^12^ to quantitatively assess the covalent resonance stabilization energy associated with the delocalization of σ-bond in the Th_3_^10+^ ring, Δ*E*^cov^_RE_ (Fig. 1a).^8^ Next, by comparison of Δ*E*^cov^_RE_ for cyclic (*D*_3h_) and linear (*D*_∞h_) isomers of Th_3_^10+^ the authors found the extra cyclic resonance energy (ECRE) to be equal 18.7 kcal mol^−1^ (Fig. 1b). This value falls between the corresponding values for the archetypical σ-aromatic H_3_^+^ (31.9 kcal mol^−1^) and non-aromatic Li_3_^+^ (0.2 kcal mol^−1^), which prompted Lin and Mo to make the conclusion that the tri-thorium ring is considerably σ-aromatic; for comparison, the reported aromatic stabilization energy in thiophene is about 18.6 kcal mol^−1^.^13^

Unfortunately, the definition of ECRE (Fig. 1a) by Lin and Mo, and the resulting prediction of aromatic stabilization energy suffer from several important issues. First, the authors assume that electronic structures of cyclic (*D*_3h_) and linear (*D*_∞h_) isomers of Th_3_^10+^ are predominated by the resonance of covalent forms. However, even a cursory look at the shapes of HOMOs in both isomers reveals that ionic forms prevail over the covalent ones with about 84–91% of charge localized on the central Th atoms. Consequently, the calculated resonance stabilization energy of 21.22 kcal mol^−1^ represents the effect of charge polarization rather than σ-bonding delocalization, which makes it not directly comparable with the covalent resonance energy of the cyclic isomer (39.88 kcal mol^−1^). Thus, the energy gap of 18.7 kcal mol^−1^ calculated by Lin and Mo cannot be strictly interpreted as the ECRE. Second, this particular result cannot be strictly interpreted as the σ-aromatic stabilization energy since it comes down with dramatic and systematic overestimation of the PBC effect.^9^ This is because the total lengths of the linear (*L* = 2 × *R*_Th–Th_) and cyclic (*L* = 3 × *R*_Th–Th_) isomer of Th_3_^10+^ are different (as well as the total number of Th–Th bonds), which prevents direct assessment of the effect of PBC on their relative stability.^9^ Lin and Mo, being aware of this potential issue, also compared the resonance energies in the cyclic (*D*_3h_) form of Th_3_^10+^ with the linear (*D*_∞h_) form of Th_4_^14+^, and found the ECRE to be equal 28.8 kcal mol^−1^.^8^ But, despite having the same number of thorium–thorium bonds, the internal potential from nuclei experienced by delocalized electrons in both cases is fundamentally different making the calculated covalent resonance stabilization energies incomparable.

## The charge separation issue in Th_3_

Aside from the issues associated with the definition of ECRE, the aromaticity predictions made for such extremely ionized model clusters like Th_3_Cl_6_^4+^ and Th_3_^10+^ may be not directly transferable to the tri-thorium core in the isolated crystalline cluster. This is because separation of charge between the Th_3_Cl_6_ cage and the cycloocta-1,3,5,7-tetraene (COT) ligands affects energy levels in the linear and cyclic isomers to a different extent, and, as will be shown in the following, the magnitude of the extra cyclic delocalization energy increases proportionally with the overall charge of the system. Moreover, the calculated covalent bond order^14^ between each Th atom and the COT ligand is equal to 0.96, thus marking sharing of the 7s^2^ electrons (Th) with the π-system of COT as an important component of chemical bonding along with strong electrovalent interactions.

**Fig. 2:**
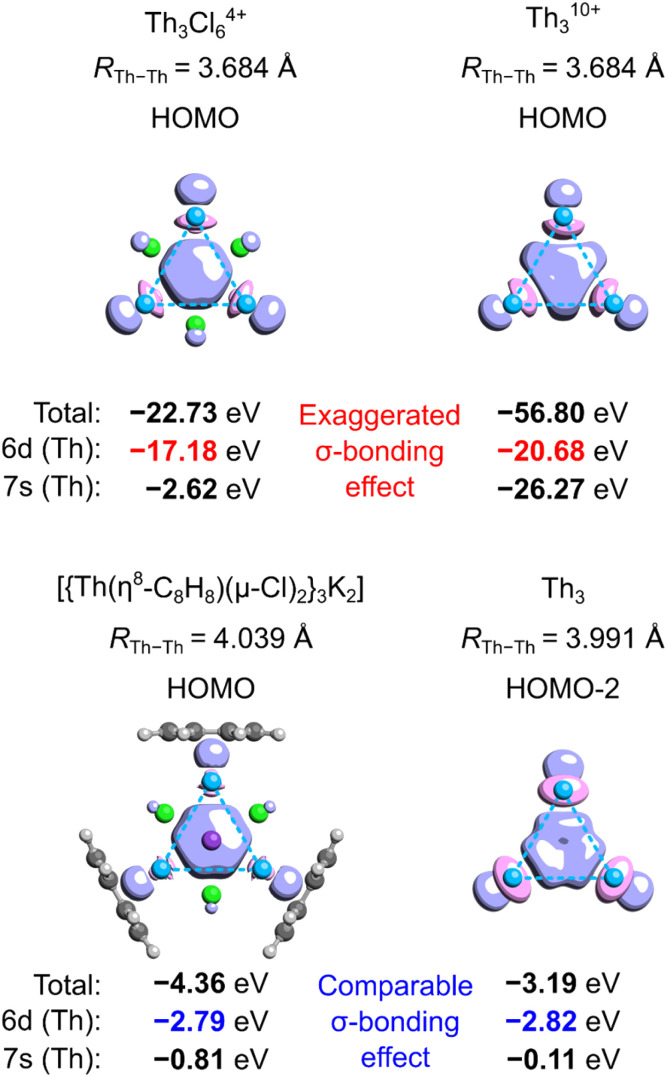
Isosurfaces (*τ* = 0.07*e*) of the 3c2e molecular orbitals in selected charged and neutral model tri-thorium clusters with the corresponding total one-electron energies as well as the energy contributions from 6d and 7s orbitals (Th). Method: ωB97X/jorge-TZP-DKH with the Douglas–Kroll–Hess 2nd-order Hamiltonian.

To illustrate to what extent separation of charge affects the tri-thorium bonding in the core of the crystalline cluster, we performed the symmetry-constrained relativistic calculations of the 3c2e-orbital energy levels in Th_3_^10+^, Th_3_Cl_6_^4+^, Th_3_, and the original model by investigated by Liddle and co-workers (Fig. 2).^3^ It should be explained at this point that in the neutral cluster Th_3_ the interference of the 3c2e σ-type HOMO−2 (−3.19 eV) with a pair of the degenerate HOMO and HOMO−1 (−2.79 eV) does not affect the resulting distribution of charge (*D*_3h_ symmetry), and, more importantly, it has no effect on the electron binding in the tri-thorium core. Indeed, a unitary transformation of the Kohn–Sham orbitals HOMO−2, HOMO−1, and HOMO into the natural bond orbitals (NBO)^15^ representation leads a subset of 3 doubly-occupied and fully localized (2c2e) σ-type bonds with the NBO energies equal to the original HOMO−2 energy level (−3.19 eV). This fact, as well as the observations reported below, justify the use of HOMO−2 in the neutral Th_3_ as an interesting alternative to the charged models of the tri-thorium bonding investigated by Lin and Mo.^8^

The results of relativistic calculations clearly show that energy levels associated with the three-center two-electron Th_3_ σ-bonding in charged clusters are essentially different from the corresponding MO energies in the neutral ones. Moreover, orbitals in charged systems are much more inhomogeneous due to significant contribution of the atomic orbitals 7s (especially in Th_3_^10+^). Also, despite visual similarity between HOMOs in Th_3_Cl_6_^4+^ and the model by Liddle and co-workers,^3^ the overlap of three 6d orbitals in the former is associated with about six times higher electron binding energy. This results in significantly shorter Th–Th bond lengths (3.684 Å) compared to the average value from the experiment (3.991 Å) and calculations by Liddle and co-workers (4.039 Å).^3^ Thus, it is clear that high ionization in the cationic Th_3_ models result in dramatic exaggeration of bonding strength, which make them hardly comparable with the experimentally validated model of the crystalline cluster.^3^ Consequently, the results of theoretical investigations involving Th_3_^10+^ and Th_3_Cl_6_^4+^ cannot be used to justify σ-aromaticity in the isolated actinide cluster.

## Aromatic stabilization from first principles

**Fig. 3:**
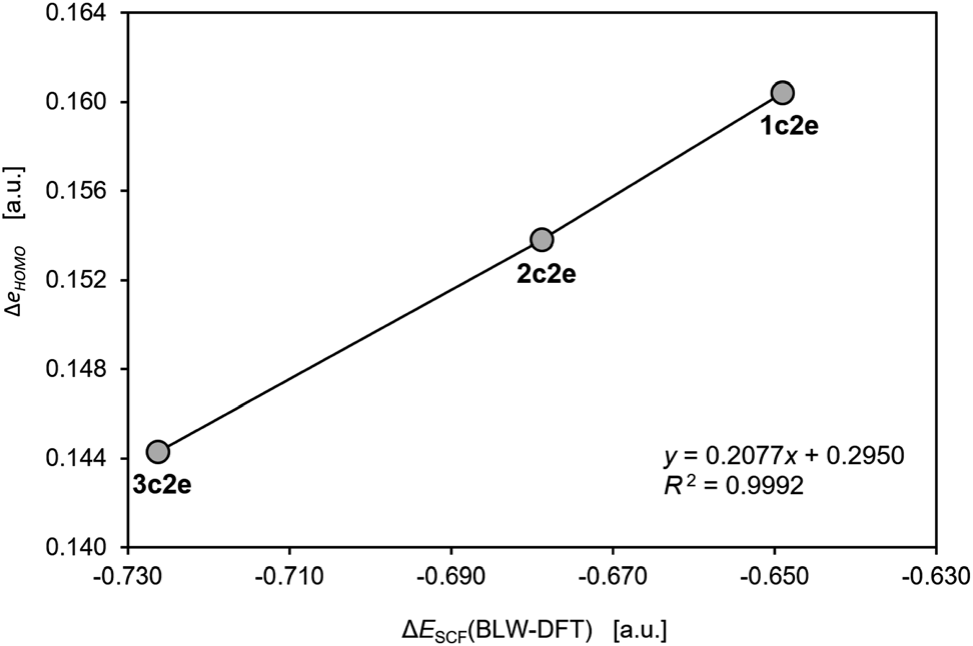
The correlation of the changes in the one-electron HOMO energy and the total energy from the self-consistent field calculations with the BLW constraints in the model cluster Th_3_^10+^. The calculations were performed using the same methods and software as used in the original work by Lin and Mo.^8^

**Fig. 4:**
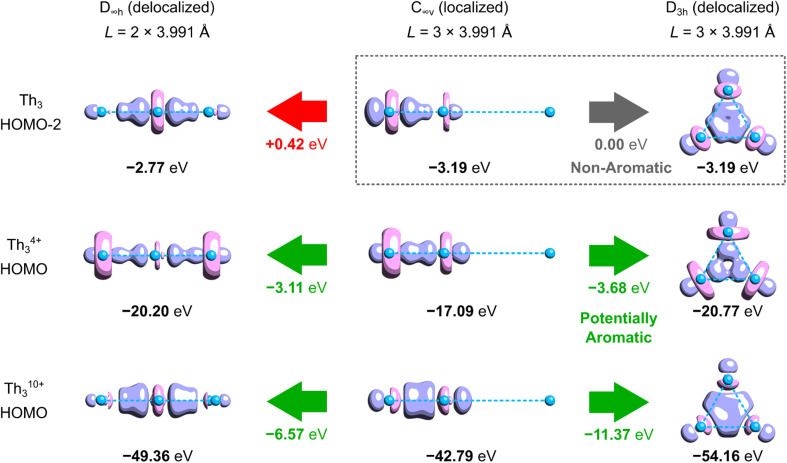
Comparison of the absolute and relative KS-MO energy levels in selected cyclic and acyclic isomers of the simplified neutral and charged tri-thorium clusters at the experimental geometry (*R*_Th–Th_ = 3.991 Å). Method: ωB97X/jorge-TZP-DKH with the Douglas–Kroll–Hess 2nd-order relativistic Hamiltonian.

Although, to the best of our knowledge, there is no reliable and universal method to quantitatively assess the effect of aromatic stabilization in all-metal clusters, the three-center two-electron bonding is a particularly special case in which stabilizing effects of the periodic boundary conditions on the electron distribution can be derived directly from the MO energy diagrams for linear and cyclic isomers. This is mainly because within the 1-electron approximation the effect of PBC on such 3c2e bonding is directly proportional to the corresponding effect on the 3c2e-type MO itself (Fig. 3). Accordingly, by comparison of the MO energy levels of the equal-length linear (*C*_∞v_) and cyclic (*D*_3h_) forms of Th_3_, Th_3_^4+^, and Th_3_^10+^, it can easily be found that only in the cases of ionic clusters the isomers with cyclically delocalized charge have orbital energies lower than the *C*_∞v_ isomers (with the electrons shared only by two thorium atoms) (Fig. 4). This means that only the charged model clusters can be considered effectively stabilized by the PBC, and hence feature aromatic stabilization.^9^ Contrariwise, in the neutral model cluster Th_3_ the corresponding 3c2e orbital in the cyclic isomer clearly makes no profits from the PBC as it has the same orbital energy as its linear counterpart with much more localized charge. It should be emphasized that the orbital energy differences between isomers *C*_∞v_ and *D*_3h_ (0.00 eV, −3.68 eV, and −11.37 eV) correlate linearly (*R*^2^ = 0.993) with the corresponding total charges of the model tri-thorium clusters (0, +4, +10), while lack of correlation is observed for the corresponding orbital energy differences between isomers *D*_∞h_ and *D*_3h_. This is a direct manifestation of the foregoing incompatibility of these isomers in the context of assessment of the PBC effects due to different total lengths, *L*.^9^ Nevertheless, the fact that localized isomer *C*_∞v_ has much lower orbital energy than the contracted isomer *D*_∞h_ with more extended charge delocalization clearly shows that the 6d atomic orbitals are reluctant to σ-conjugation at this distance. This, in turn, is in full agreement with the conclusions from our previous study.^7^

**Fig. 5:**
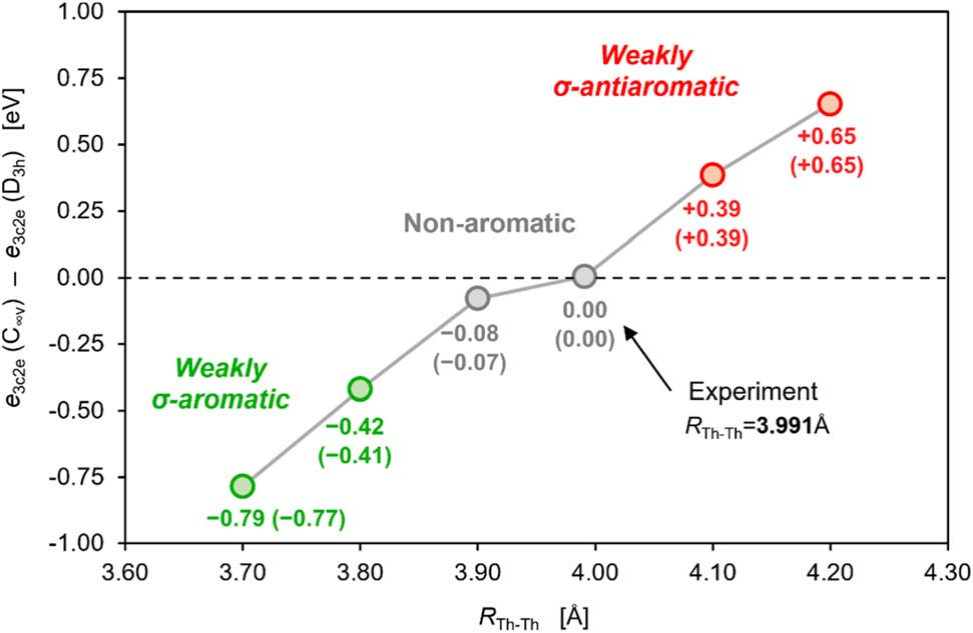
The stabilizing and destabilizing effects of the PBC on the 3c2e σ-bonding in the neutral model cluster Th_3_ at different bond lengths, *R*_Th–Th_. Method: ωB97X/jorge-TZP-DKH with the Douglas–Kroll 2nd-order relativistic Hamiltonian (numbers in brackets refer to the corresponding relativistic calculations including the spin–orbit coupling).

It should also be noted that at distances shorter than 3.991 Å the overlap of 6d orbitals in the neutral tri-thorium core would be more effective, and weak but noticeable σ-aromatic stabilization could be theoretically possible (Fig. 5). However, one must realize that even a small contraction of the Th–Th bond length in the isolated crystalline actinide cluster implies size reduction of the entire Th_3_Cl_6_ cage and the resulting enhancement of the Pauli repulsion between lone pairs of chlorine atoms crowed in the relatively small volume. This has been argued to have a negative impact on thermodynamic stability of the entire cluster.^7^ Thus, the strong multicenter charge-shift bonding in the Th_3_Cl_6_ cage seems to stretch the small ring of weakly bonded thorium atoms to the extent at which the extra cyclic stabilization is no more effective.

## Conclusions

The crystalline tri-thorium cluster represents an interesting and important case in which cyclic delocalization of electrons inside the all-metal core is not associated with aromatic stabilization, but is dictated by high symmetry of the network of surrounding Th–Cl bonds.^7^ In fact, the charge transfer between thorium and chlorine atoms makes the multicenter charge-shift bonding in the entire Th_3_Cl_6_ cage particularly strong.^8^ But, at the same time it impairs the direct σ-bonding between thorium atoms to the extent at which the Th_3_ ring exhibits no extra cyclic stabilization compared to its linear and more localized isomer (*C*_∞v_). Thus, in accordance with the very first sentence of the recommendation by IUPAC,^11^

*‘Aromaticity is the concept of spatial and electronic structure of cyclic molecular systems displaying the effects of cyclic electron delocalization which provide for their enhanced thermodynamic stability relative to acyclic structural analogues (…)’*

the tri-thorium core in the isolated crystalline cluster is non-aromatic. Similar conclusion was made by Foroutan-Nejad and co-workers who investigated the magnetic-response properties of the model tri-thorium cluster at the fully relativistic level of the theory.^6^ Interestingly, it has been demonstrated that the oxidation of the original cluster to its counterpart without the characteristic 3c2e-type HOMO noticeably enhances the charge-shift bonding in the Th_3_Cl_6_ cage (shorter Th–Cl bonds), which almost doubles the HOMO–LUMO gap.^7^ This means that the cyclic delocalization of electrons in the Th_3_ ring may affect thermodynamic stability of the entire complex thus putting into question the core–shell syngenetic model proposed by Lin and Mo.^8^ It should be noted that the proposed methodology to assess the ASE directly from the MO energy levels is limited to the three-membered rings investigated at the one-determinant (HF or DFT) theory level provided that the corresponding 3c2e-type MO does not effectively interfere with other orbitals in the MO spectrum (which is the case in the neutral Th_3_). Moreover, the predicted vanishing ASE may still be subject to some uncertainty since the proposed simplified model clusters (to a limited extent) suffer from the charge separation issue (the Th_3_ core in the isolated cluster is not neutral).^3,10^ Nevertheless, the presented results clearly show that aromatic stabilization in Th_3_ increases linearly with the total charge of the system, and therefore the results of investigations involving highly ionized model clusters cannot be used to justify σ-aromatic character of the isolated crystalline cluster.

## Methodology

The extra cyclic resonance energy calculations were performed using the same methods and software as utilized in the original investigation by Lin and Mo.^8^ The input files (as well as details on implementation of the BLW-DFT code) were kindly provided by Dr Lin., and they are available from the author on request. All the molecular geometries of the model tri-thorium clusters were taken from the original article by Liddle and co-workers^3^ and the recently published paper by Lin and Mo.^8^ The relativistic calculations were performed with Gaussian G16 (rev. C01)^16^ and the implemented therein DKH2-relativistic Hamiltonian;^17^ for the neutral Th_3_ model cluster the spin–orbit coupling (SOC) effects were included in the calculations at different *R*_Th–Th_*via* the keyword DKHSO, but the results shown rather marginal effect on the calculated energy gaps (Fig. 5). The exchange-correlation functional ωB97X (ref. 18) by the Head-Gordon group was used to get reliable description of the one-electron energy levels, especially the frontiers ones, which are of crucial importance for the presented study. In this context it should be noticed that ωB97X has demonstrated that performs excellent in reproduction of the experimental ionization potentials for the aromatic systems of different types within the framework of the DFT-Koopman's theorem.^19^ In all relativistic calculations the all-electron triple-zeta valence basis set developed by Jorge and co-workers was used.^20^ It should be noted that other all-electron basis sets available for Th were found to suffer from significant Rydberg contributions to the ground-state density matrix by inclusion of orbitals with larger azimuthal quantum numbers (8s, 6f, *etc.*), which is unphysical and leads to exaggeration of bonding density between thorium atoms; a more comprehensive examination of the role of the choice of the basis set and the exchange-correlation functional on the description of bonding in actinide clusters will be the subject of a separate study. The SuperFine grid and very tight convergence criteria were used in the self-consistent field (SCF) calculations. To calculate the covalent bond orders and estimate the degree of charge localization in all the model Th_3_ clusters the linear coefficients of natural atomic orbitals were used from the NBO7 program (the keyword NAOMO in the $NBO section).^15^ The original code of the BLW-DFT method has been modified to facilitate direct calculations of the PBC effects; the modified BLW-DFT code is available on request from the author.

## Conflicts of interest

There are no conflicts to declare.

## Supplementary Material
